# Prescription of blood pressure lowering treatment after intracerebral haemorrhage: Prospective, population-based cohort study

**DOI:** 10.1177/2396987320975724

**Published:** 2020-12-03

**Authors:** Karl Bonello, Amy PK Nelson, Tom J Moullaali, Rustam Al-Shahi Salman, for the Lothian Audit of the Treatment of Cerebral Haemorrhage Collaborators

**Affiliations:** 1 Department of Clinical Neurosciences, Royal Infirmary of Edinburgh, Edinburgh, UK; 2 Institute of Neurology, UCL, London, UK; 3 University of Edinburgh Medical School, 47 Little France Cres, Edinburgh, UK; 4 Centre for Clinical Brain Sciences, University of Edinburgh, Edinburgh, UK; 5 The George Institute for Global Health, Faculty of Medicine, University of New South Wales, NSW, Australia

**Keywords:** Intracerebral haemorrhage, blood pressure, secondary prevention, population-based study

## Abstract

**Introduction:**

Blood pressure (BP) lowering reduces the risk of recurrent stroke after intracerebral haemorrhage (ICH). However, implementation of BP lowering in clinical practice in the UK is unknown.

**Patients and methods:**

We identified adults with first-ever incident ICH to quantify the proportion who survived >14 days after hospital discharge and were prescribed BP-lowering medication in a prospective, population-based, inception cohort study in the Lothian region of Scotland during June 2010–May 2012 and January–December 2019. After the first cohort, we analysed reasons for avoiding BP-lowering medication in a sample from the Lothian region of the Scottish Stroke Care Audit during January 2017–November 2017, which informed a quality improvement intervention that was implemented in the second cohort.

**Results:**

After efforts to improve monitoring and lowering of BP amongst ICH survivors, there was an increase in the proportion of patients prescribed BP-lowering medication at hospital discharge between the first and second population-based cohorts (81/130 [62%] vs. 68/89 [76%]; P = 0.028). Compared with patients not prescribed BP-lowering medication at hospital discharge, patients prescribed BP-lowering medication presented with higher systolic BP (177 vs. 156 mm Hg, P = 0.002 and 180 vs. 149 mm Hg, P < 0.001, in the first and second population-based cohorts, respectively), and were more likely to have pre-morbid hypertension (85% vs. 33%, P < 0.001 and 72% vs. 29%, P < 0.001) and atrial fibrillation (35% vs. 4%, P < 0.001 and 26% vs. 5%, P < 0.034).

**Conclusion:**

In this population-based study, the proportion of patients with ICH who were prescribed BP-lowering medication at hospital discharge increased after a quality improvement intervention.

## Introduction

Elevated blood pressure (BP) is the strongest modifiable risk factor for intracerebral haemorrhage (ICH) and accounts for approximately half of the population-attributable risk.^1,2^ In patients who survive beyond 90 days after ICH, failure to achieve adequate BP control is common,^3^ and is associated with a higher risk of recurrent lobar and non-lobar ICH,^4^ and other adverse cardiovascular outcomes.^5^

In 2004, the perindopril protection against recurrent stroke study (PROGRESS) randomised controlled trial showed that BP lowering reduced the relative risk of recurrent stroke by 50% in ICH survivors.^6^ The United Kingdom national stroke guidelines were updated in 2012 to recommend lowering systolic BP to <130 mm Hg in stroke survivors where there are no contraindications.^7^

We sought to determine the frequency of prescription of BP-lowering medication and reasons for avoidance in ICH survivors at hospital discharge in the Lothian region of Scotland, United Kingdom. Our findings informed a quality improvement intervention that aimed to increase the use of BP monitoring and BP-lowering medication in Lothian. We compared the proportion of ICH survivors who were prescribed BP-lowering medication at hospital discharge in a population-based cohort study before and after the quality improvement intervention to identify a temporal change in practice, and assessed patient characteristics associated with the prescription of BP-lowering medication to identify areas for further improvement.

## Methods

### Setting

#### Prospective, population-based, longitudinal cohort study of ICH used for audit, research and quality improvement

We identified patients with ICH (presumed due to cerebral small vessel disease) in The Lothian Audit of the Treatment of Cerebral Haemorrhage (LATCH) from 1 June 2010 to 31 May 2012, and 1 January 2019 to 31 December 2019. LATCH ascertained cases from the Lothian Health board region of Scotland (including 3 hospitals) in residents aged ≥16 years diagnosed with first-ever or recurrent ICH (mid-2010 population aged ≥16 years: 695,335) through multiple routes: prospective hot pursuit sources through a trust-wide network of physicians, neurologists, neurosurgeons, radiologists, pathologists, specialist nurses and audit personnel, daily neuroradiology meetings and review of all computed tomographic (CT) head scans; and retrospective searches of electronic patient notes, International Classification of Diseases 10th Revision (ICD-10) coded hospital discharge records (http://www.isdscotland.org), the Scottish Stroke Care Audit (http://www.strokeaudit.scot.nhs.uk), and records at the Office of the Procurator Fiscal.^8^

#### Retrospective search of the Scottish Stroke Care Audit (SSCA) database

In order to inform a quality improvement intervention that could be evaluated during the second population-based study, we did a retrospective search of the Scottish Stroke Care Audit (SSCA) database to identify patients with ICH in Lothian from 2 January 2017 to 9 November 2017, and examined their hospital records to identify reasons why BP-lowering medication was not prescribed at hospital discharge.

#### Study participants

We defined ICH as a symptomatic event (new headache, focal neurological symptoms, or altered consciousness), referable to a discrete parenchymal bleed (confirmed by temporally consistent radiology or pathology findings). We included patients with incident first-ever ICH presumed due to cerebral small vessel disease who survived >14 days after hospital discharge. We reviewed patients’ medical records and investigations to exclude cases of recurrent ICH, isolated extra-axial intracranial haemorrhage, and ICH definitely attributable to a macrovascular cause, tumour, trauma, and haemorrhagic transformation of ischaemic stroke. Standard clinical practice in NHS Lothian involves targeted use of CT angiography informed by clinical predictors of the risk of underlying macrovascular pathology.^9^

#### LATCH audit standard

The LATCH audit standard relating to long-term BP lowering after ICH is as follows: ‘*blood pressure-lowering therapy should be prescribed by hospital discharge if systolic BP is >130 mm Hg, unless there is a contra-indication to the use of these drugs (e.g. end of life care, hypotension et*c).’ Therefore, the primary outcome was the prescription of BP-lowering medication at hospital discharge, irrespective of prior BP-lowering medication use or prior hypertension diagnosis.

#### Quality improvement intervention

The 2017 audit indicated that there was a persisting unexplained deficit in proportion of ICH survivors that received a prescription for BP-lowering medication at hospital discharge, so we sought to improve the availability and use of BP monitoring and BP-lowering medication where it was indicated by clinical guidelines^7,10^ by implementing a quality improvement intervention on 1 November 2018 (https://services.nhslothian.scot/Stroke/ClinicalAudits/LATCH%20protocol.pdf). The intervention involved the introduction of Lothian-wide guidance on the treatment of ICH (Acute stroke due to intracerebral haemorrhage: Assessment, Consultation and Treatment [ICH-ACT]), and the opportunity for ICH survivors to use a home BP tele-monitoring service (Florence tele-monitoring [https://www.getflorence.co.uk/]) or participate in one of two ongoing research studies of BP monitoring and BP lowering after ICH (Triple Therapy Prevention of Recurrent Intracerebral Disease EveNts Trial [TRIDENT, NCT02699645] and Prevention of Hypertensive Injury to the Brain by Intensive Treatment in IntraCerebral Haemorrhage [PROHIBIT-ICH, NCT03863665]).

To promote the quality improvement intervention, we organised a series of education sessions about ICH-ACT and the various BP monitoring and lowering activities for key stakeholders in secondary care (colleagues in neurology, stroke, acute internal medicine and emergency departments). We worked closely with clinical colleagues to maintain engagement with the intervention: this included weekly departmental meetings attended by stroke clinicians, and monthly email reminders to LATCH collaborators (supplementary material). We anticipated that these activities would improve the uptake of guideline-recommended BP management by raising awareness of this important intervention amongst patients and their hospital-based healthcare practitioners, and by providing several options for their long-term BP management.^7,10^

#### Data collection

We retrospectively collected prospectively recorded data on BP-lowering medication use after ICH from paper and electronic patient records associated with the corresponding hospital admission including initiation of, or changes to, BP-lowering medication during admission and BP-lowering prescription on discharge. In the population-based cohorts, we extracted additional data from patients’ medical records: medical history; use of BP-lowering, antiplatelet and anticoagulant medication at ICH diagnosis; clinical data on admission (BP and Glasgow Coma Scale score); and haematoma characteristics on baseline CT brain scan (anonymised scans were prospectively collected and held on a secure server). We rated ICH location on baseline CT brain scans according to the CHARTS rating system.^11^ In view of prescribing patterns in the first population-based cohort, we assessed reasons for the avoidance of BP-lowering medication in the audit and the second population cohort (these data were not available for the first population-based cohort). To assess participation in several BP-monitoring activities available from 2018 onwards, we collected information about the long-term BP monitoring plan from the electronic discharge summary in the second population cohort.

#### Statistical analysis

To identify a temporal change in practice, we compared the proportion of ICH survivors who were prescribed BP-lowering medication at hospital discharge before (first population-based cohort) and after (second population-based cohort) the quality improvement intervention using the chi-squared test. We assessed the associated change in the proportion of ICH survivors who were prescribed BP-lowering at hospital discharge in a multivariable logistic regression model adjusted for clinically important baseline confounders, which included age (≥80 years vs. <80 years), Glasgow coma scale (GCS) score (≤12 vs. >12), ICH location (infratentorial vs. other), presence of intraventricular haemorrhage, presence of any cardiovascular comorbidity, and prior use of anticoagulation. We also compared the two population-based cohorts by their patient characteristics, stratified by prescription of BP-lowering medication at hospital discharge (yes vs. no), using the Kruskal-Wallis test for continuous variables, and the chi-squared test for categorical variables. The significance level was set at P = 0.05.

## Results

**Figure 1.**
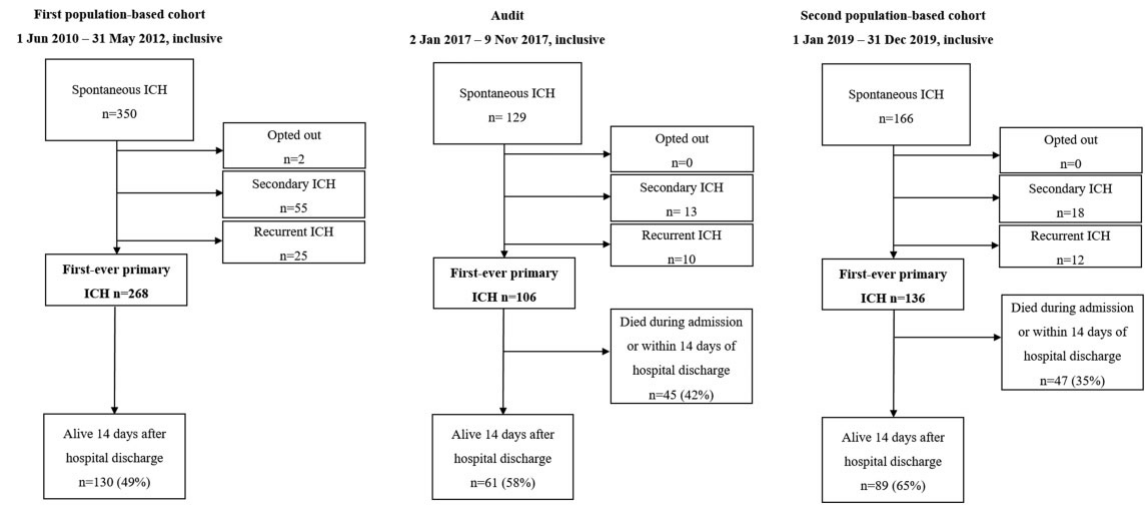
Flowchart of patients included in the study. Percentages relate to those with first-ever primary ICH. First population-based cohort denotes the study period 1 June 2010 to 31 May 2012 inclusive; audit, 2 January 2017 to 9 November 2017; second population-based cohort, 1 January 2019 to 31 December 2019; ICH, intracerebral haemorrhage.

**Figure 2.**
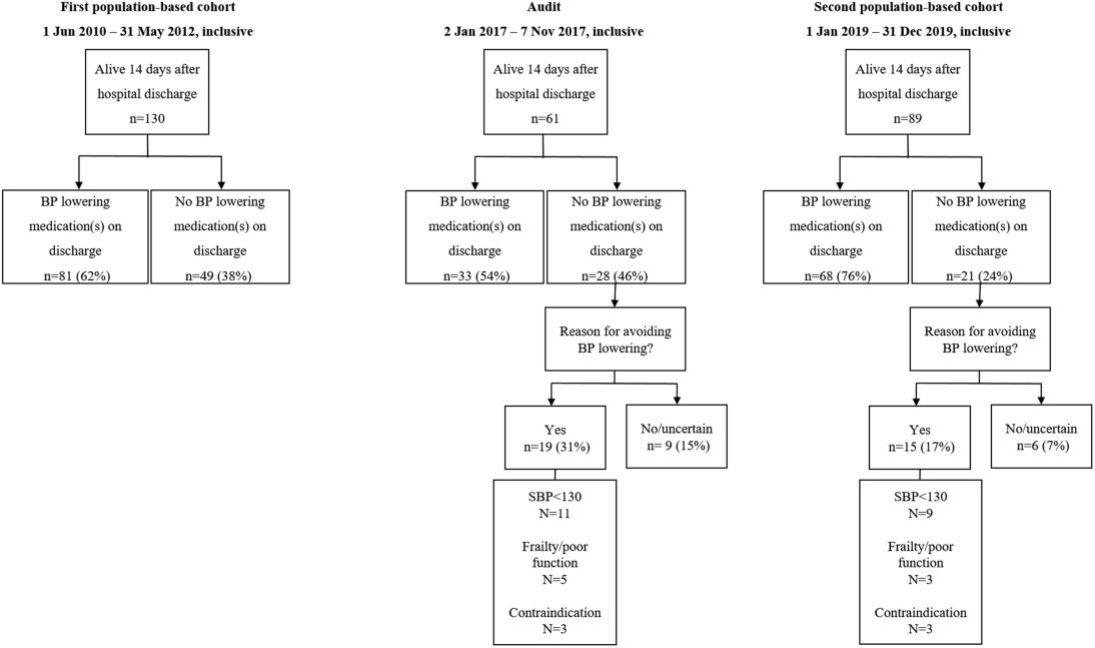
Proportion of ICH patients who survived more than 14 days after hospital discharge and received a prescription for BP-lowering medication, and reasons for avoidance, by study epoch. Percentages relate to those with first-ever primary ICH who were at alive at 14 days after hospital discharge. First population-based cohort denotes the study period 1 June 2010 to 31 May 2012 inclusive; audit, 2 January 2017 to 9 November 2017; second population-based cohort, 1 January 2019 to 31 December 2019; ICH, intracerebral haemorrhage; BP, blood pressure; *SBP < 130, patients achieving systolic BP levels <130 mm Hg without BP-lowering medication*.

In the first population-based cohort (1 June 2010 to 31 May 2012, inclusive), there were 268 cases of incident first-ever ICH of whom 130 (49%) survived >14 days after hospital discharge (Figure 1). Among 130 ICH patients who survived >14 days after hospital discharge, 81 (62%) were prescribed BP-lowering medication (Figure 2).

The subsequent audit that informed the quality improvement intervention (2 January 2017 to 9 November 2017, inclusive) revealed that of 61 patients with ICH who survived >14 days after hospital discharge, 33 (54%) were prescribed BP-lowering medication, 19 (31%) were not prescribed BP-lowering medication and had a reason for avoiding them and 9 (15%) were not prescribed BP-lowering medication and did not have a clear reason for avoiding them (Figure 2).

Following the introduction of the quality improvement intervention, the second population-based cohort (1 January 2019 to 31 December 2019, inclusive) revealed that of 136 cases of incident first-ever ICH, 89 (65%) survived >14 days after hospital discharge. A comparison of the baseline characteristics of the first and second population-based cohorts is shown in Supplementary Table 1: compared to the first population-based cohort, anticoagulant use was more common, and subarachnoid extension less common in the second population-based cohort. Among 89 patients with ICH who survived >14 after from hospital discharge, 68 (76%) patients were prescribed BP-lowering medication, 15 (17%) patients were not prescribed BP-lowering medication and had a reason for avoiding them, and 6 (7%) patients were not prescribed BP-lowering medication and did not have a clear reason for avoiding them (Figure 2). Compared with the proportion of patients who received BP-lowering medication at hospital discharge in the first population-based cohort, the proportion of patients who received BP-lowering medication in the second population-based cohort represented a statistically significant increase (76% vs. 62%, P = 0.028). In adjusted analyses, the association between the quality improvement intervention and the proportion of patients prescribed BP-lowering medication at hospital discharge persisted (adjusted odds ratio 2.26 [95% confidence interval 1.08 to 4.73], p = 0.030, area under ROC curve 0.81).

The distribution of reasons for avoiding BP-lowering medication was similar in the audit and second population-based cohort: among patients with a reason for avoiding BP-lowering, there were 11/19 (58%) and 9/15 (60%) with systolic BP levels <130 mm Hg; 5/19 (26%) and 3/15 (20%) with frailty/poor function; and 3/19 (16%) and 3/15 (20%) with a contraindication to BP-lowering medication in the audit and second population-based cohort, respectively.

Table 1 shows the baseline characteristics of ICH patients who survived >14 days after hospital discharge, stratified by population-based cohort epoch and prescription of BP-lowering medication at hospital discharge. Compared with patients not prescribed BP-lowering medication at hospital discharge, patients prescribed them were similar in age and sex, and the proportion with deep/infratentorial haematoma location was also similar (57% vs. 46%, P = 0.241 and; 57% vs. 48%, P = 0.433, in the first and second population-based cohorts, respectively).

**Table 1.** Baseline characteristics of patients with incident first-ever intracerebral haemorrhage who survived >14 days after hospital discharge, stratified by population-based study epoch and prescription of BP-lowering medication at hospital discharge.

	First population-based cohort			Second population-based cohort		
	BP lowering at hospital discharge		P	BP lowering at hospital discharge		P
	Yes(N = 81)	No(N = 49)		Yes(N = 68)	No(N = 21)	
Age at onset, years	74 (59–80)	74 (61–82)	0.792	74 (62–82)	74 (57–82)	0.674
Gender, female	44 (54)	29 (59)	0.588	31 (46)	10 (48)	0.870
Medical history						
Hypertension	69 (85)	16 (33)	<0.001	48 (72)	6 (29)	<0.001
Atrial fibrillation	28 (35)	2 (4)	<0.001	18 (26)	1 (5)	0.034
Myocardial infarction	8 (10)	1 (2)	0.088	3 (4)	0 (0)	0.327
Ischaemic stroke	11 (14)	5 (10)	0.570	7 (10)	2 (10)	0.918
Transient ischaemic attack	7 (9)	1 (2)	0.129	4 (6)	0 (0)	0.255
Diabetes mellitus	8 (10)	2 (4)	0.230	13 (19)	1 (5)	0.114
Peripheral vascular disease	3 (4)	0 (0)	0.173	1 (1)	1 (5)	0.374
Hyperlipidaemia	18 (22)	11 (22)	0.976	13 (19)	0 (0)	0.030
Medications at admission^a^						
BP-lowering			<0.001			<0.001
None	23 (28)	45 (92)		24 (36)	18 (86)	
One	20 (25)	3 (6)		16 (24)	3 (14)	
Many	38 (47)	1 (2)		27 (40)	0 (0)	
Antiplatelet			0.018			0.786
None	44 (54)	38 (78)		53 (79)	16 (76)	
One	33 (41)	11 (22)		13 (19)	5 (24)	
Many	4 (5)	0 (0)		1 (2)	0 (0)	
Anticoagulant			0.003			0.041
None	68 (84)	49 (100)		50 (75)	20 (95)	
One	13 (16)	0 (0)		17 (25)	1 (5)	
Clinical assessment						
Systolic BP, mm Hg	177 (38)	156 (27)	0.002	180 (33)	149 (27)	<0.001
Diastolic BP, mm Hg	95 (25)	85 (18)	0.060	97 (24)	83 (15)	0.027
GCS score	15 (14-15)	14 (13-15)	0.031	15 (14-15)	15 (14-15)	0.298
Haematoma characteristics						
Location^b^			0.241			0.433
Deep/infratentorial	43 (57)	21 (46)		39 (57)	10 (48)	
Lobar	33 (43)	25 (54)		29 (34)	11 (52)	
Intraventricular extension	25 (33)	14 (30)	0.778	20 (29)	4 (20)	0.350
Subarachnoid extension	25 (33)	20 (43)	0.240	14 (21)	4 (20)	0.878
Subdural extension	4 (5)	4 (9)	0.458	1 (1)	1 (5)	0.374

Data are number (%), mean (standard deviation) or median (interquartile range). First population-based cohort denotes the study period 1 June 2010 to 31 May 2012 inclusive; second population-based cohort, 1 January 2019 to 31 December 2019.

^a^Missing data for 1 patient in epoch three initially admitted to a hospital in another health board.

^b^CHARTS rating: 6 patients in epoch three were rated ‘uncertain – holohemispheric’ and are not included.

Compared with patients not prescribed BP-lowering medication at hospital discharge, patients prescribed them presented with higher systolic BP (177 vs. 156 mm Hg, P = 0.002 and; 180 vs. 149 mm Hg, P < 0.001, in the first and second population-based cohorts, respectively), and were more likely to have pre-morbid hypertension (85% vs. 33%, P < 0.001 and; 72% vs. 29%, P < 0.001) and atrial fibrillation (35% vs. 4%, P < 0.001 and; 26% vs. 5%, P < 0.034), and be prescribed BP-lowering medication (none vs. one vs. many: P < 0.001 and P < 0.001) and anticoagulant medication (none vs. one: P = 0.003 and P = 0.041) at symptom onset. Overall, there were 48/130 (37%) and 19/89 (21%) prescribed antiplatelet medication, and 13/130 (10%) and 18/89 (20%) prescribed anticoagulant medication at the time of ICH onset in the first and second population-based cohorts, respectively.

Table 2 shows the BP management of ICH patients: 3 or medications were prescribed in 26/128 (20%) and 21/89 (24%) of patients in the first and second population-based cohorts, respectively; 8/128 (6%), and 7/89 (8%) of patients received the combination of a renin-angiotensin system blocker, calcium channel blocker and diuretic. In the first population-based cohort and the audit, renin-angiotensin system blockers were the most frequently prescribed agent, compared with the second population-based cohort where calcium channel blockers were used most frequently. In the second population-based cohort, long-term BP monitoring was attributed to the patient’s general practitioner in 41/89 (46%) patients, home tele-monitoring in 10/89 (11%) patients, home self-monitoring in 3/89 (3%) patients and a clinical trial in 3/89 (3%) patients; no plan was documented in 32/89 (36%) patients.

**Table 2.** Blood pressure management of patients with ICH at hospital discharge.

	First population-based cohort (N = 128^a^)	Audit(N = 61)	Second population-based cohort (N = 89)
Number of BP-lowering medications			
0	49 (38)	28 (46)	21 (24)
1	32 (25)	19 (31)	26 (29)
2	21 (16)	9 (15)	21 (24)
≥3	26 (20)	5 (8)	21 (24)
Median	1 (0–2)	1 (0–1)	1 (1–2)
BP-lowering medication class			
Calcium channel blocker	25 (20)	13 (21)	43 (48)
Renin-angiotensin system blocker	49 (38)	17 (28)	41 (46)
Diuretic	29 (23)	8 (13)	15 (17)
Beta-adrenoreceptor blocker	28 (22)	11 (18)	30 (34)
Alpha-adrenoceptor blocker	7 (5)	3 (5)	6 (7)
Nitrate	17 (13)	2 (3)	3 (3)
Other	0 (0)	0 (0)	1 (1)
Combination therapy			
RAS blocker + CCB + diuretic	8 (6)	0 (0)	7 (8)
BP-monitoring plan			
GP	–	–	41 (46)
None	–	–	32 (36)
Home tele–monitoring	–	–	10 (11)
Home self–monitoring	–	–	3 (3)
Clinical trial	–	–	3 (3)

First population-based cohort denotes the study period 1 June 2010 to 31 May 2012 inclusive; audit, 2 January 2017 to 9 November 2017; second population-based cohort, 1 January 2019 to 31 December 2019; BP, blood pressure; RAS, renin–angiotensin system; CCB, calcium channel blocker; GP, general practitioner.

^a^Two patients in epoch one were prescribed BP–lowering at hospital discharge, but details were unavailable.

## Discussion

In two epochs of this prospective, population-based, longitudinal cohort study and an interim audit, between half and three quarters of ICH survivors were prescribed BP-lowering medication at hospital discharge; around a third of patients who did not receive BP-lowering medication did not have a clear reason for avoiding them. In 2019 (the second population-based cohort), there was a significant increase in the proportion of patients who received BP-lowering medication to 76% from 62% in the first population-based cohort; 24% were prescribed 3 or more agents, and the use of calcium channel blockers overtook renin-angiotensin system blockers. Premorbid hypertension and atrial fibrillation were more common among patients prescribed BP-lowering treatment at hospital discharge compared with patients who were not; age, sex and the proportion of patients with deep/infratentorial haematoma location were similar.

Despite evidence of benefit emerging from the PROGRESS trial nearly two decades ago,^6,12^ and updates to the UK National Clinical Guidelines for Stroke in 2012^7^ and European Stroke Organisation guidelines in 2014,^10^ prescription of BP-lowering medication for the secondary prevention of recurrent stroke after ICH could be further improved. Yet, while little change occurred between 2010–2012 (first population-based cohort) and 2017 (audit), we found an increase in the proportion of ICH patients that received BP-lowering at hospital discharge in 2019 (second population-based cohort), which was associated with the introduction of a quality improvement intervention involving Lothian-wide guidance on ICH management and opportunities to recruit patients to two randomised controlled trials (TRIDENT and PROHIBIT-ICH) and a home BP tele-monitoring initiative (Florence) from 2018 onwards. These findings are encouraging, and may reflect increasing awareness of the benefits of BP-lowering after ICH among clinicians due to embedded quality improvement.

Our findings are similar to those from other observational studies of prescribing practice after ICH: 74% of patients were prescribed BP-lowering medication after ICH in a recent, large Australian study,^13^ 61% in a large US study^4^; and when considered alongside all-cause strokes, varying but substantial under-prescribing is reported in Canada^14^ and the UK.^15^ To understand patient factors that influence prescribing habits, we stratified patients by population-based study epoch and BP-lowering medication prescription at hospital discharge. We found pre-morbid hypertension and atrial fibrillation were more common among patients who received BP lowering in first and second population-based cohorts, and hyperlipidaemia was more common in patients who received BP lowering in the second population-based cohort; differences in prior myocardial infarction, transient ischaemic attack, and diabetes mellitus did not meet statistical significance. These findings are consistent with those from an Australian registry where detailed comorbidity data were collected in 1,498 ICH patients,^13^ and when considered together, probably reflect the continued use of pre-morbid BP-lowering medication in patients with a diagnosis of hypertension or other cardiovascular comorbidity, and β-blockers prescribed for atrial fibrillation. The PROGRESS trial results showed that benefits from BP-lowering treatment are likely to exist irrespective of baseline BP and other cardiovascular comorbidities.^6,12^ Therefore, it is important to reach all patients without a contraindication to BP-lowering medication.

By the same criteria, we assessed differences in admission BP and haematoma characteristics on diagnostic CT scan: compared with patients who were not prescribed BP-lowering medication at hospital discharge, patients prescribed them had significantly higher BP on admission to hospital. We also identified that a systolic BP level <130 mm Hg was a common reason for avoiding BP-lowering medication. This indicates some degree of concordance with clinical guidelines,^10,16^ where BP lowering to a systolic BP target <140 mm Hg is recommended during the first 7 days due the potential for modest benefits on function and health-related quality of life,^17^ followed by continued treatment to achieve systolic BP levels <130 mm Hg in the long term. However, haematoma location, and presence of extension into intraventricular, subarachnoid and subdural spaces did not significantly differ according to discharge BP lowering. These findings are encouraging in view of evidence to suggest benefits from long-term BP lowering exist in both lobar and non-lobar ICH,^4,18^ although our small sample size may have precluded the detection of any true differences.

A key methodological strength of this study is its population-based design: to our knowledge, all incident ICH cases in the Lothian population were captured through multiple sources of ascertainment. Patient characteristics associated with the prescription of BP-lowering medication at hospital discharge were internally consistent in two population-based cohorts separated by nearly a decade. These similarities suggest that changes in the proportion of patients who were prescribed BP-lowering medication may have resulted from the introduction of quality improvement measures rather than changes in patient characteristics. Other temporal changes in prescribing appeared to reflect emerging evidence, including: more frequent use of calcium channel blockers at hospital discharge where data suggest greater efficacy for stroke prevention^19,20^; and more frequent use of anticoagulants in patients with atrial fibrillation, where reductions in the risk of ischaemic stroke are greater than in patients taking antiplatelets.^21^ However, our small sample and multiple comparisons increase the likelihood of chance findings.

We acknowledge there are limitations to our study. First, the increase in the proportion of patients with ICH who were prescribed BP-lowering at hospital discharge associated with the quality improvement intervention is prone to confounding from unmeasured factors, including temporal changes in practice related to early intensive BP lowering. Second, in the absence of long-term follow up data from patients identified by our population-based study in 2019, we were unable to assess temporal changes in the level of concordance with treatment in the community, nor the proportion achieving optimal systolic BP control in the longer term. In view of our finding that multi-agent treatment regimens are common among ICH survivors, and recent evidence to suggest a fixed low-dose polypill in addition to standard care is safe, tolerable and lowers BP more than standard care alone in middle-aged adults with hypertension,^22^ randomised evidence from the TRIDENT randomised controlled trial is awaited to determine the effects of this strategy on recurrent stroke and vascular events in ICH survivors.

In summary, the prescription of BP-lowering medication at hospital discharge for the prevention of recurrent stroke after ICH over the last decade could be improved upon. A quality improvement intervention that involved Lothian-wide guidance on the management of ICH, and the opportunity for ICH survivors to participate in several BP monitoring and BP-lowering activities, was associated with an increase in the proportion of patients who received BP-lowering medication at hospital discharge. Further improvements may be possible if BP lowering is considered in patients without a previous diagnosis of hypertension or other cardiovascular comorbidities.

## Supplemental Material

sj-pdf-1-eso-10.1177_2396987320975724 - Supplemental material for Prescription of blood pressure lowering treatment after intracerebral haemorrhage: Prospective, population-based cohort study Click here for additional data file. Supplemental material, sj-pdf-1-eso-10.1177_2396987320975724 for Prescription of blood pressure lowering treatment after intracerebral haemorrhage: Prospective, population-based cohort study by Karl Bonello, Amy PK Nelson, Tom J Moullaali, Rustam Al-Shahi Salman and for the Lothian Audit of the Treatment of Cerebral Haemorrhage Collaborators in European Stroke Journal

## References

[1] Rapsomaniki E Timmis A George J, et al. Blood pressure and incidence of twelve cardiovascular diseases: lifetime risks, healthy life-years lost, and age-specific associations in 1·25 million people. Lancet 2014; 383: 1899–1911. 24881994 10.1016/S0140-6736(14)60685-1 PMC4042017

[2] O'Donnell MJ Chin SL Rangarajan S, et al. Global and regional effects of potentially modifiable risk factors associated with acute stroke in 32 countries (INTERSTROKE): a case-control study. Lancet 2016; 388: 761–775. 27431356 10.1016/S0140-6736(16)30506-2

[3] Zahuranec DB Wing JJ Edwards DF, et al. Poor long-term blood pressure control after intracerebral hemorrhage. Stroke 2012; 43: 2580–2585. 22903494 10.1161/STROKEAHA.112.663047 PMC3458127

[4] Biffi A Anderson CD Battey TWK, et al. Association between blood pressure control and risk of recurrent intracerebral hemorrhage. J Am Med Assoc 2015; 314: 904–912. 10.1001/jama.2015.10082 PMC4737594 26325559

[5] Kim J Bushnell CD Lee HS, et al. Effect of adherence to antihypertensive medication on the long-term outcome after hemorrhagic stroke in Korea. Hypertension 2018; 72: 391–398. 29915019 10.1161/HYPERTENSIONAHA.118.11139

[6] Chapman N Huxley R Anderson C, et al. Effects of a perindopril-based blood pressure-lowering regimen on the risk of recurrent stroke according to stroke subtype and medical history: the PROGRESS trial. Stroke 2004; 35: 116–121. 14671247 10.1161/01.STR.0000106480.76217.6F

[7] Royal College of Physicians. *Intercollegiate stroke working party: National Clinical Guidelines for Stroke*. 4th ed. London: 2012.

[8] Samarasekera N Fonville A Lerpiniere C, et al. Influence of intracerebral hemorrhage location on incidence, characteristics, and outcome: population-based study. Stroke 2015; 46: 361–368. 25586833 10.1161/STROKEAHA.114.007953

[9] Hilkens NA Van Asch CJJ Werring DJ, et al. Predicting the presence of macrovascular causes in non-traumatic intracerebral haemorrhage: the DIAGRAM prediction score. J Neurol Neurosurg Psychiatry 2018; 89: 674–679. 29348301 10.1136/jnnp-2017-317262

[10] Steiner T Al-Shahi Salman R Beer R, et al. European Stroke Organisation (ESO) guidelines for the management of spontaneous intracerebral hemorrhage. Int J Stroke 2014; 9: 840–855. 25156220 10.1111/ijs.12309

[11] Charidimou A Schmitt A Wilson D, et al. The Cerebral Haemorrhage Anatomical RaTing inStrument (CHARTS): development and assessment of reliability. J Neurol Sci 2017; 372: 178–183. 28017207 10.1016/j.jns.2016.11.021

[12] MacMahon S Neal B Tzourio C, et al. Randomised trial of a perindopril-based blood-pressure-lowering regimen among 6105 individuals with previous stroke or transient ischaemic attack. Lancet 2001; 358: 1033–1041. 11589932 10.1016/S0140-6736(01)06178-5

[13] Dalli LL Kim J Thrift AG, et al. Disparities in antihypertensive prescribing after stroke linked data from the Australian Stroke Clinical Registry. Stroke 2019; 50: 3592–3599. 31648630 10.1161/STROKEAHA.119.026823

[14] Mouradian MS Majumdar SR Senthilselvan A, et al. How well are hypertension, hyperlipidemia, diabetes, and smoking managed after a stroke or transient ischemic attack? Stroke 2002; 33: 1656–1659. 12053007 10.1161/01.str.0000017877.62543.14

[15] Johnson P Rosewell M James MA. How good is the management of vascular risk after stroke, transient ischaemic attack or carotid endarterectomy? Cerebrovasc Dis 2007; 23: 156–161. 17124397 10.1159/000097053

[16] Hemphill JC Greenberg SM Anderson CS, et al. Guidelines for the management of spontaneous intracerebral hemorrhage. Stroke 2015; 46: 2032–2060. 26022637 10.1161/STR.0000000000000069

[17] Anderson C Heeley E Huang Y, et al. Rapid blood-pressure lowering in patients with acute intracerebral hemorrhage. N Engl J Med 2013; 368: 2355–2365. 23713578 10.1056/NEJMoa1214609

[18] Arima H Tzourio C Anderson C, et al. Effects of perindopril-based lowering of blood pressure on intracerebral hemorrhage related to amyloid angiopathy: the progress trial. Stroke 2010; 41: 394–396. 20044530 10.1161/STROKEAHA.109.563932

[19] Rothwell PM Howard SC Dolan E, et al. Effects of beta blockers and calcium-channel blockers on within-individual variability in blood pressure and risk of stroke. Lancet Neurol 2010; 9: 469–480. 20227347 10.1016/S1474-4422(10)70066-1

[20] Webb AJ Fischer U Mehta Z, et al. Effects of antihypertensive-drug class on interindividual variation in blood pressure and risk of stroke: a systematic review and meta-analysis. Lancet 2010; 375: 905–915. 10.1016/S0140-6736(10)60235-8 20226989

[21] Hart RG Pearce LA Aguilar MI. Meta-analysis: antithrombotic therapy to prevent stroke in patients who have nonvalvular atrial fibrillation. Ann Intern Med 2007; 146: 857–867. 17577005 10.7326/0003-4819-146-12-200706190-00007

[22] Webster R Salam A De Silva HA, et al. Fixed low-dose triple combination antihypertensive medication vs usual care for blood pressure control in patients with mild to moderate hypertension in Sri Lanka a randomized clinical trial. J Am Med Assoc 2018; 320: 566–579. 10.1001/jama.2018.10359 PMC6583010 30120478

