# Supplementary material for: Prescription of blood pressure lowering treatment after intracerebral haemorrhage: Prospective, population-based cohort study
Source: Eur Stroke J. 2020 Dec 3;6(1):44–52. doi: 10.1177/2396987320975724 (PMC7995321; doi:10.1177/2396987320975724)
Supplement: sj-pdf-1-eso-10.1177_2396987320975724 - Supplemental material for Prescription of blood pressure lowering treatment after intracerebral haemorrhage: Prospective, population-based cohort study [file sj-pdf-1-eso-10.1177_2396987320975724.pdf]

Supplementary appendix

Prescription of blood pressure lowering treatment after intracerebral haemorrhage: prospective, population-based cohort study

Karl Bonello MD□[1] Amy P. K. Nelson MD□,[2,3] Tom J. Moullaali MBBS□,[4,5] Rustam Al-Shahi Salman PhD[4] for the Lothian Audit of the Treatment of Cerebral Haemorrhage Collaborators*

[1]Royal Infirmary of Edinburgh, 51 Little France Cres, Edinburgh, UK

[2]Institute of Neurology, UCL, London, UK.

[3]University of Edinburgh Medical School, 47 Little France Cres, Edinburgh, UK

[4]Centre for Clinical Brain Sciences, University of Edinburgh, University of Edinburgh, Edinburgh, UK

[5]The George Institute for Global Health, Faculty of Medicine, University of New South Wales, NSW, Australia

□Contributed equally

Corresponding author:

Professor Rustam Al-Shahi Salman

Centre for Clinical Brain Sciences

Chancellor's Building

49 Little France Crescent

Edinburgh

EH16 4SB

Email: rustam.al-shahi@ed.ac.uk

Supplementary Table 1 Baseline characteristics of patients with incident first-ever intracerebral haemorrhage who survived >14 days after hospital discharge, stratified by population-based study epoch

	Population-based cohort		
	First (N=130)	Second (N=89)	P
Age at onset, years	74 (60-81)	74 (61-82)	0.694
Gender, female	73 (56)	41 (46)	0.142
**Medical history**			
Hypertension	85 (65)	54 (61)	0.545
Atrial fibrillation	30 (23)	19 (21)	0.763
Myocardial infarction	9 (7)	3 (3)	0.257
Ischaemic stroke	16 (12)	9 (10)	0.616
Transient ischaemic attack	8 (6)	4 (4)	0.596
Diabetes mellitus	10 (8)	14 (16)	0.061
Peripheral vascular disease	3 (2)	2 (2)	0.977
Hyperlipidaemia	29 (22)	13 (15)	0.155
**Medications at admission***			
BP-lowering			0.726
None	68 (52)	42 (47)	
One	23 (18)	19 (22)	
Many	39 (30)	27 (31)	
Antiplatelet			0.051
None	82 (63)	69 (78)	
One	44 (34)	18 (21)	
Many	4 (3)	1 (1)	
Anticoagulant			0.030
None	117 (90)	70 (80)	
One	13 (10)	18 (20)	
**Clinical assessment**			
Systolic BP, mm Hg	169 (36)	173 (34)	0.349
Diastolic BP, mm Hg	91 (23)	94 (23)	0.405
GCS score	15 (14-15)	15 (14-15)	0.300
**Haematoma characteristics**			
Location^			0.709
Deep/infratentorial	64 (52)	49 (55)	
Lobar	58 (48)	40 (45)	
Intraventricular extension	39 (32)	24 (27)	0.433
Subarachnoid extension	45 (37)	18 (20)	0.009
Subdural extension	8 (7)	2 (2)	0.146

Data are number (%), mean (standard deviation) or median (interquartile range)
First population-based cohort denotes the study period 1 June 2010 to 31 May 2012 inclusive; second population-based cohort, 1 January 2019 to 31 December 2019
*Missing data for 1 patient in epoch three initially admitted to a hospital in another health board
^CHARTS rating: 6 patients in epoch three were rated ‘uncertain – holohemispheric’ and are not included

Lothian Audit of Treatment of Cerebral Haemorrhage (LATCH) collaborators

Karen Adamson, Anne Addison, Kate Ahmad, Mukhtar Ahmed, Syed Alhadad, Judith Anderson, Peter Andrews, Ganesh Arunagirinathan, Andrew Baird, Laura Barr, Amanda Barugh, Caroline Bates, Nicola Bell, Elaine Bisset, Gordon Blair, Tom Blankenstein, Peter Bodkin, Friederike Boellert, Ralph Bouhaidar, Paul Brennan, James Bridson, Seona Broadbent, John Brush, Laura Butler, Dave Caesar, Brian Campbell, Patricia Cantley, Jonathan Carter, Ana Casado, Sarah Chambers, Siddharthan Chandran, Una Clancy, Gareth Clegg, Peter Connick, Helen Cook, Phillip Copley, Andrew Coull, Rebecca Cranfield, Alastair Crosswaite, Olimpia Curran, Vera Cvora, Richard Davenport, James Dear, Andreas Demetriades, Martin Dennis, Chris Derry, Sandra Dewar, Katrina Dodds, Fergus Doubal, John Downer, Simon Dummer, Susan Duncan, Fiona Duncan, Johannes DuPlessis, Katy Easterford, Andrew Elder, Trish Elder-Gracie, Sarah Eljamel, Kevin Enright, Kate Enright, Martin Errington, Morgan Evans, Fiona Ewing, Donald Farquhar, Tom Fitzgerald, Alastair Fitzgerald, Michael Fitzpatrick, Peter Foley, Jon Foley, Jane Fothergill, Ioannis Fouyas, Brian Frier, Pasquale Gallo, Sudipto Ghosh, Rod Gibson, Helen Gillett, John Gilmour, Stephen Glancy, Clive Goddard, Claire Gordon, Jim Gordon-Smith, Robin Grant, David Grant, Alasdair Gray, Ali Harmouche, Lianne Harrison, Simon Hart, Chris Hay, Robin Henderson, Russell Hewett, Mark Hughes, Fiona Hughes, David Hunt, Neil Hunter, Aidan Hutchison, Hamish Ireland, James Ironside, Alan Jaap, Simon Jackson, Katherine Jackson, Ashok Jacob, Andrew Jamieson, Alan Japp, Mark Jones, Anne Jones, Michael Jones, Colin Josephson, Chandru Kaliaperumal, Anant Kamat, Jothy Kandasamy, Susan Kealey, Sarah Keir, Gillian Kerr, Simon Kerrigan, Peter Keston, Sonnie Khan, Matthew King, Ravin Kishen, Richard Knight, Anne Knox, Peter Lange, Simon Leigh-Smith, Chris Lerpiniere, Imran Liaquat, James Loan, Ann Lockman, Jane Lumsden, Elizabeth Macdonald, Graham Mackay, Graeme Mackenzie, Michael Mackenzie, Donald MacLeod, Malcolm Macleod, Conor Maguire, Grant Mair, Steven Makin, Neil Masson, Moyra Masson, Ashok Mathews, Simon Maxwell, Fiona Maxwell, Gashirai Mbizvo, Jon McCafferty, Lynn McCallum, Stuart McClellan, Simon McGurk, Andrew McIntosh, Martin McKechnie, Chris Anne Mckenzie, Scott McKie, Graham McKillop, John McKnight, Siobhan McLaughlin, Suzanne McLenachan, Malcolm Mcleod, Emily McMurray, Gillian Mead, Tracey Millar, Fiona Minns, Gillian Moffat Pickering, mireia

moragas, Fiona Moreton, Wendy Morley, Zoe Morris, Lewis Morrison, Billie Morrow, Frank Morrow, Tim Morse, Sam Moultrie, John Muchison, Kenneth Muir, Colin Mumford, Latana Munang, Jon Murchison, Rachael Murphy, Lee Murphy, Ross Murphy, Katherine Murray, Lynn Myles, Hafid Narayan, Alison Nelson, Yi Ng, Jasmine Ng, Graham Nimmo, Donald Noble, Richard O'Brien, Fiona O'Brien, Olayinka Ogundipe, Suvankar Pal, Ruwan Parakrama, Dilip Patel, Fergus Perks, Alison Pollock, Michael Poon, Mohd Noh Radzi, Scott Ramsay, Kristiina Rannikmae, Matthew Reed, Phil Reid, Jo Renton, Jonathan Rhodes, Jane Rimer, Gillian Ritchie, Geraint Roberts, Sara Robinson, Mark Rodrigues, Jerard Ross, Tim Russell, Waleed Salih, Neshika Samarasekera, Peter Sandercock, Euan Sandilands, Shona Scott, Robin Sellar, Johann Selvarajah, Mano Shanmuganathan, Joanne Sharkey, Himanshu Shekhar, Henry Simms, Johanna Simpson, Mara Sittampalam, Randy Smith, Tim Soane, Drahus Sokol, Hamza Soleiman, Linda Spence, Helen Spiers, Patrick Statham, Neo Stavrinos, Andy Stevenson, Gareth Stewart, Claire Stirling, Jon Stone, Mark Strachan, Joyce Stuart, Cathie Sudlow, David Summers, Rachel Sutherland, Vicky Tallentire, John Taylor, Pat Taylor, Bethany Threlfall, Iain Todd, Antonia Torgersen, Neil Turner, Margarethe van Dijke, Edwin Van Beek, Liesbeth Van Look, Akila Visvanathan, Andrew Walker, Robert Walker, Tim Walsh, Joanna Wardlaw, Frauke Weidanz, Graeme Weir, Belinda Weller, Mark White, William Whiteley, Ian Whittle, Anthony Wiggins, Tim Wilkinson, Robert Will, Andrew Williams, Matthew Wilson, James Wilson, Charis Wong, Rebecca Woodfield, Julie Woodfield, Rohana Wright, Stanko Yordanov, Ruth Young, Wendy Young.
